# Supplementary material for: One Year of Recombinant Human Growth Hormone Treatment in Adults with Prader–Willi Syndrome Improves Body Composition, Motor Skills and Brain Functional Activity in the Cerebellum
Source: J Clin Med. 2022 Mar 25;11(7):1831. doi: 10.3390/jcm11071831 (PMC8999376; doi:10.3390/jcm11071831)
Supplement: Supplementary file 1 [file jcm-11-01831-s001.zip › jcm-1619253-supplementary.pdf]

Supplementary Materials and Methods

Functional MRI Testing

Patients with Prader–Willi syndrome (PWS) underwent two identical functional magnetic resonance imaging (fMRI) scan sessions on separate days, at baseline and 12 months after GH treatment initiation. On both MRI scanning days, participants were given instructions before the scan concerning fMRI testing procedures and the need to remain still during acquisition. Twenty-three patients with PWS had valid fMRI assessments at baseline and 22 patients had valid fMRI assessments at 12 months.

Motor Tests during fMRI

Functional MRI testing involved the performance of three manual tasks of different motor complexity [1,2]: (1) Repetitive flexion-extension of one hand. Participants were required to make repetitive self-paced opening and closing motions of the hand at a trained rate of one flexion-extension cycle every two seconds, alternating hands for 30 s each, beginning with the right hand; (2) bimanual anti-phase repetitive flexion-extension movements. This task consisted of flexion and extension of the two hands with a phase shift of 180 between them (one hand flexes while the other extends); and (3) repetitive sequence of fingers-to-thumb opposition movements with the right hand. Participants were instructed to consecutively connect the touch balls of the thumb with the other fingers in a complex self-paced sequence beginning with the index finger, followed by the middle finger, the ringer finger, and finally the little finger.

Functional MRI Acquisition

A Philips Achieva 3.0 Tesla magnet equipped with an eight-channel phased-array head coil and single-shot echo planar imaging (EPI) software (version 5.3, Philips Healthcare, Best, The Netherlands) was used for the fMRI assessment. The functional sequences consisted of gradient recalled acquisition in the steady state (time of repetition (TR), 2000 ms; time of echo (TE), 35 ms; pulse angle, 70) within a field of view of 230 x 230 mm, with a 64 x 64-pixel matrix, and a slice thickness of 3.59 mm (inter-slice gap, 0 mm). a total of 34 interleaved slices were acquired to cover the whole brain. Three 3-min scans were acquired for each participant. Each functional time series consisted of 90 consecutive image volumes obtained during each of the three-minute assessments. The first four (additional) image volumes in each run were discarded to allow magnetization to reach equilibrium.

For each of the three fMRI tests, we used an identical 3AB block-design paradigm consisting of six 30 s blocks, totaling 3 min in duration, which corresponded to three blocks of 30 s of right hand movement alternating with three blocks of 30 s of left-hand movement in the first task, and to three rest (baseline) blocks of 30 s alternating with three movement blocks of 30 s in the second and third tasks. The examiner visually controlled task performance and gave the commands “now move” and “now pause” at the beginning and end of the respective hand movement blocks. During the rest period, participants were asked to simply lie still and not think about hand movements.

Functional MRI Preprocessing

Imaging data were processed using MATLAB version 2016a (The MathWorks Inc, Natick, Mass) and Statistical Parametric Mapping (SPM12; The Wellcome Department of Imaging Neuroscience, London). Preprocessing involved motion correction, spatial normalization, and smoothing by means of a gaussian kernel of full-width half-maximum 8 mm. Data were normalized to the standard SPM-EPI template and resliced to 2 mm isotropic resolution in Montreal Neurological Institute (MNI) space. All image sequences were visually inspected for potential acquisition and normalization artifacts.

Control of Potential head Motion Effects

To control for the effects of head motion, we adopted the following approach: (i) time series were aligned to the first functional image (of each time series) in each participant using a least squares minimization and a six-parameter (rigid body) spatial transformation. (ii) We included six motion-related regressors in the first-level (single subject) analyses. (iii) Within-subject, censoring-based MRI signal artifact removal (scrubbing) [3] was used to discard motion-affected volumes. For each participant, mean inter frame motion measurements [4] served as an index of data quality to flag volumes of suspect quality across the run. At points with mean inter frame motion >0.3 mm, we discarded the corresponding volume and the succeeding volume. Using this procedure, a mean (±SD; range) of 7.6 (±9.5; 0–30) volumes were removed for the first task analysis, 6.4 (±7.5; 0–24) volumes for the second task, and 5.0 (±5.3; 0–17) volumes for the third task analysis at baseline [5]. Volume removal for each task at 12 months was 3.4 (±5.8; 0–25), 3.2 (±4.6; 0–13), and 4.6 (±6.9; 0–18), respectively. (iv) A minimum of 120 s (60 volumes) with no motion artifacts after scrubbing was required for participants to be included in the analyses. As a result of this criteria, from the initial sample of 22 subjects with valid fMRI assessments at both baseline and 12 months, data from two participants were removed in the simple task analysis, and data from another two participants were removed in the analysis of the bimanual task. (v) The remaining potential motion effects were controlled by including a motion summary measurement for each participant as a covariate in the group analyses in SPM [4].

Functional MRI Analysis

To obtain individual maps of brain activity evoked during motor challenge, a boxcar regressor was generated considering the three blocks of the baseline condition and the three blocks of the test condition, and applying a hemodynamic delay of 4 s. Contrast "right hand < left hand" and "right hand > left hand" images were estimated for each participant in the unimanual flexion-extension task. The contrasts "baseline < motion" (activation) and "baseline > motion" (deactivation) were estimated instead for both the bimanual anti-phase flexion-extension and the finger-to-thumb opposition tasks. Resulting first-level SPM contrast images for each subject were carried forward to group-level random-effects analyses. One-sample *t*-test designs were used to generate group activation maps at 12 months, and paired *t*-tests were used to compare brain activation (baseline *vs* 12 months) for each of the three motor tests, including data from 20 participants in the first and second task analyses and 22 participants in the third task analysis.

In addition, to identify the brain areas in which the magnitude of the activation changes was related to the change in the clinical parameters, we estimated voxel-wise the correlations between brain activation changes (i.e., 12 months minus baseline values) and clinical changes by using linear regression in SPM. This analysis was limited to the clinical variables showing significant within-group differences (i.e., TUG and BBS scores, IFG-1, and percentage of fat and lean mass). For each subject, "difference" images were computed over 12 months and baseline conditions and the images were used to map the activation changes in the whole sample by means of one sample *t*-test.

In all analyses, results were considered significant with clusters > 2.3 mL (290 voxels) at a height threshold of $p < 0.005$, which satisfied the family-wise error (FWE) rate correction of $p_{FWE} < 0.05$, according to recent Monte-Carlo simulations [5].

References

1. Martino J, Gabarrós A, Deus J, Juncadella M, Acebes JJ, Torres A, Pujol J. Intrasurgical mapping of complex motor function in the superior frontal gyrus. *Neuroscience*. 2011, *179*,131–142.
2. Pujol J, Conesa G, Deus J, López-Obarrio L, Isamat F, Capdevila A. Clinical application of functional magnetic resonance imaging in presurgical identification of the central sulcus. *J Neurosurg*. 1998, *88*, 863-869.
3. Power JD, Mitra A, Laumann TO, Snyder AZ, Schlaggar BL, Petersen SE. Methods to detect, characterize, and remove motion artifact in resting state fMRI. *Neuroimage*. 2014, *84*,320–341.

4. Pujol J, Macià D, Blanco-Hinojo L, Martínez-Vilavella G, Sunyer J, de la Torre R, Caixàs A, Martín-Santos R, Deus J, Harrison BJ. Does motion-related brain functional connectivity reflect both artifacts and genuine neural activity? *Neuroimage*. 2014 *101*, 87–95.
5. Blanco-Hinojo L, Casamitjana L, Pujol J, Martínez-Vilavella G, Esteba-Castillo S, Giménez-Palop O, Freijo V, Deus J, Caixàs A. Cerebellar dysfunction in adults with Prader Willi syndrome. *J Clin Medicine*. 2021,*10*,3320.
